# Monocarboxylate transporter-1 promotes osteoblast differentiation via suppression of p53, a negative regulator of osteoblast differentiation

**DOI:** 10.1038/s41598-018-28605-5

**Published:** 2018-07-12

**Authors:** Kiyohito Sasa, Kentaro Yoshimura, Atsushi Yamada, Dai Suzuki, Yoichi Miyamoto, Hiroko Imai, Kazuhiro Nagayama, Koutaro Maki, Matsuo Yamamoto, Ryutaro Kamijo

**Affiliations:** 1 Departments of Biochemistry, Showa University School of Dentistry, Tokyo, Japan; 2 Departments of Periodontology, Showa University School of Dentistry, Tokyo, Japan; 3 Departments of Orthodontics, Showa University School of Dentistry, Tokyo, Japan

## Abstract

Monocarboxylate transporter-1 (MCT-1) is a transmembrane transporter for monocarboxylates including lactate and pyruvate. Silencing *Mct1* by its small interfering RNA (siRNA) suppressed the expression of marker genes for osteoblast differentiation, namely, *Tnap*, *Runx2*, and *Sp7*, induced by BMP-2 in mouse myoblastic C2C12 cells. *Mct1* siRNA also suppressed alkaline phosphatase activity, as well as expressions of *Tnap* and *Bglap* mRNAs in mouse primary osteoblasts. On the other hand, *Mct1* siRNA did not have effects on the Smad1/5 or ERK/JNK pathways in BMP-2-stimulated C2C12 cells, while it up-regulated the mRNA expression of p53 (*Trp53*) as well as nuclear accumulation of p53 in C2C12 cells in a BMP-2-independent manner. Suppression of osteoblastic differentiation by *Mct1* siRNA in C2C12 cells was abolished by co-transfection of *Trp53* siRNA. Together, these results suggest that MCT-1 functions as a positive regulator of osteoblast differentiation via suppression of p53.

## Introduction

Monocarboxylate transporters (MCTs) are a group of transmembrane transporters for monocarboxylates. Among 14 subtypes identified thus far, inward and outward transportation of H^+^ and monocarboxylates, such as lactate and pyruvate, have been shown to be mediated by 4 types of MCTs, i.e., MCT-1, -2, -3, and -4^1^. Of those, MCT-1 is ubiquitously expressed, and its major physiological role is believed to facilitate influx and efflux of L-lactate across the plasma membrane depending on the cellular metabolic state. For example, L-lactate absorbed through MCT-1 is used for gluconeogenesis in parenchymal cells of the liver and the proximal convoluted tubule cells of the kidney^2^. Furthermore, L-lactate and ketone bodies absorbed through MCT-1 are used as fuel for aerobic respiration in cardiac muscle and red skeletal muscle cells^3^. On the other hand, most cells must release L-lactate to outside of the cell via MCT-1 when glycolysis is predominant under a hypoxic condition. Transportation of H^+^ together with L-lactate through MCT-1 prevents intracellular acidification, which is preferable for maintaining an intracellular condition suitable for high glycolytic flux^3^.

In our previous study, we demonstrated that MCT-1 is required for chondrocyte death induced by interleukin-1β (IL-1β), which occurred in a nitric oxide- and reactive oxygen species (ROS)-dependent manner^4^. Furthermore, MCT-1 knockdown by siRNA strongly suppressed expression of phagocyte-type NADPH oxidase (NOX-2), an enzyme specialized for production of ROS, in mouse chondrocyte-like ATDC5 cells. We also found that an MCT-1-dependent increase in mitochondrial ROS production was required for late phase activation of NF-κB, which led to expression of NOX-2 in ATDC5 cells stimulated by IL-1β. Together, those observations suggested that MCT-1 might have possible hidden functions, in addition to regulation of energy metabolism and intracellular pH. MCT-1 is also known to be ubiquitously expressed in various tissues including bones^1,5^. Furthermore, we have shown that acetoacetate enhanced and β-hydroxybutyrate suppressed mineralization by mouse osteoblastic MC3T3-E1 cells, in both the presence and absence of bone morphogenetic protein (BMP)-2^6^. These ketone bodies are known to be transported across plasma membranes via MCT-1^1^, while knockdown of *Mct1* nullified their effects^6^.

Those findings led us to explore the role of MCT-1 in osteoblast differentiation in the present study, for which we chose osteoblastic differentiation of mouse myoblastic C2C12 cells induced by BMP-2 as a model of osteoblast differentiation^7^. BMP-2, a cytokine that belongs to the transforming growth factor-β superfamily, binds to a complex of type I and type II receptors, and induces phosphorylation and activation of Smad-1, -5, and -8, transcription factors that activate the expression of RUNX2, a master transcription factor for osteoblast differentiation, while BMP receptors also activate the MAP-kinase pathway^8^. Based on the present results, we herein describe a function of MCT-1 in BMP-2-induced osteoblast differentiation, including the Smad and MAP-kinase pathways. In addition, we also examined the role of MCT-1 in activation of p53, a negative regulator of osteoblast differentiation^9^.

## Results

### *Mct1* knockdown suppressed osteoblast differentiation of C2C12 cells after stimulation by BMP-2

In C2C12 cells, the expression level of *Mct1* mRNA was the highest among mRNAs for MCT-1, -2, -3, and -4 (Suppl. Fig. S1a), known transmembrane transporters for monocarboxylate such as lactate and pyruvate^1^. Hence we silenced *Mct1* mRNA expression by introducing *Mct1* siRNA into C2C12 cells (Suppl. Fig. S1b). Intracellular lactate in *Mct1* siRNA-introduced C2C12 cells was 3 times higher than that in cells introduced with the control siRNA (Suppl. Fig. S1c). Since intracellular lactate is thought to be generated as a result of glycolysis, its elevated concentration is assumed to be the result of suppressed efflux of lactate through MCT-1 in *Mct1* siRNA-introduced C2C12 cells.

**Figure 1:**
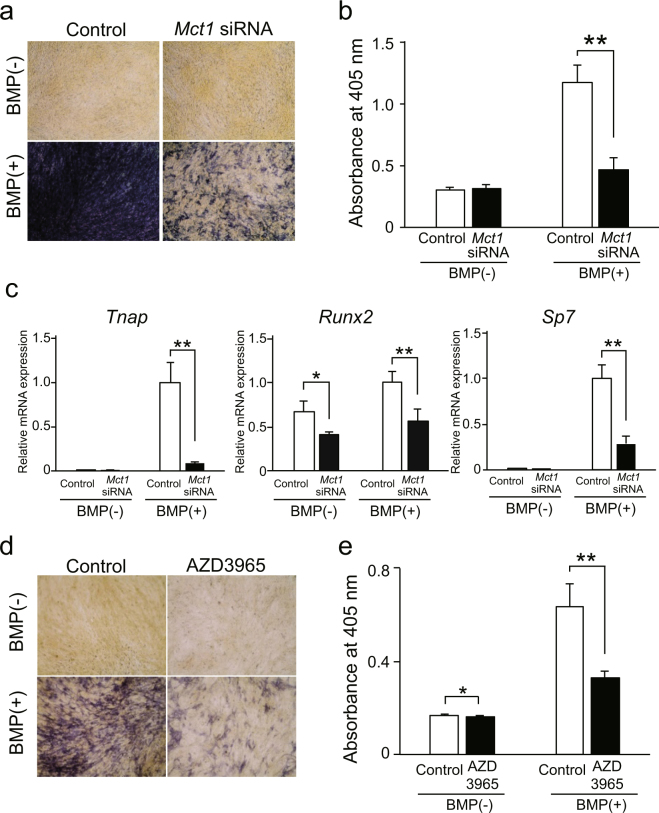
Suppression of osteoblastic differentiation of C2C12 cells by introduction of *Mct1* siRNA or inhibition of MCT-1 activity. C2C12 cells introduced with control or *Mct1* siRNA were cultured for 72 (**a**,**b**) or 48 (**c**) hours in the absence (−) or presence (+) of BMP-2 (300 ng/mL). (**a**) Results of ALP activity staining. (**b**) ALP activity in lysates of C2C12 cells. (**c**) Expressions of mRNAs for *Tnap*, *Runx2*, and *Sp7* were determined using real-time RT-PCR, then normalized against that of *Gapdh*, with the results shown as relative values. (**d**) C2C12 cells were cultured for 48 hours in the presence or absence of 1 μmol/L of AZD3965, a specific inhibitor of MCT-1, and with (+) or without (−) BMP-2 (300 ng/mL). ALP activity was detected by staining. (**e**) ALP activity in lysates of C2C12 cells cultured under the same conditions. (**b**,**c**,**e**) Values are expressed as the mean ± SD (n = 3–5). **Significantly different from control group (*p* < 0.01).

*Mct1* knockdown did not have effects on proliferation of C2C12 cells in either the presence or absence of BMP-2 (Suppl. Fig. S1d). It is conceivable that differentiation of C2C12 cells into osteoblast-like cells induced by BMP-2 mimics the differentiation of osteoblasts^10^. Hence, we analyzed the effect of *Mct1* knockdown on osteoblastic differentiation of C2C12 cells stimulated by BMP-2. We found that the increase in number of ALP-positive cells, enhanced ALP activity, and increased expression of tissue-nonspecific ALP gene (*Tnap*) in C2C12 cells after exposure to BMP-2 were each suppressed by introduction of *Mct1* siRNA (Fig. 1a–c). In addition, *Mct1* knockdown suppressed mineralization of extracellular matrices of C2C12 cells cultured in the presence of BMP-2 (Suppl. Fig. S2). Furthermore, *Mct1* knockdown suppressed BMP-2-induced expression of genes for transcription factors required for osteoblast differentiation, i.e., RUNX2 (*Runx2*) and Osterix (*Sp7*), in C2C12 cells following stimulation with BMP-2 (Fig. 1c). Also, down-regulation of ALP activity and *Tnap* expression were observed in C2C12 cells treated with AZD3965, a specific inhibitor of MCT-1 (Fig. 1d,e). These results suggest that lowered expression of MCT-1 or its inhibition suppresses BMP-2-induced differentiation of C2C12 cells into osteoblast-like cells.

### *Mct1* knockdown suppressed expression of ALP in mouse calvarial osteoblasts

**Figure 2:**
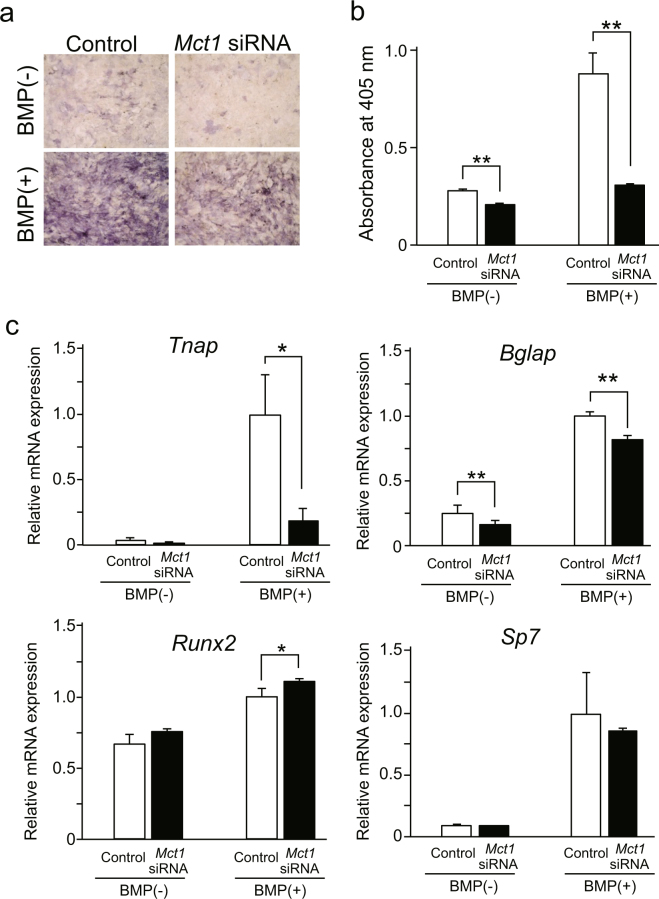
Suppressed expression of osteoblast differentiation markers in primary mouse osteoblasts by introduction of *Mct1* siRNA. Control or *Mct1* siRNA was introduced into primary osteoblasts isolated from mouse calvaria. Osteoblasts were cultured for 72 (**a**,**b**) or 48 (**c**) hours in the absence (−) or presence (+) of BMP-2 (300 ng/mL). (**a**) ALP activity, detected by staining. (**b**) ALP activity in cell lysates. (**c**) Expression of mRNAs for *Tnap*, *Bglap*, *Runx2*, and *Sp7* were analyzed by real-time RT-PCR, then normalized against that of *Gapdh*, with the results indicated as relative values. (**b**,**c**) Data are expressed as the mean ± SD (n = 3–5). *, **Significantly different from control group (*p < 0.05, **p < 0.01).

In order to investigate the role of MCT-1 in expression of osteoblast phenotypes by differentiated osteoblasts, we examined the effects of *Mct1* knockdown on ALP activity and expression of osteoblast marker genes in mouse primary osteoblasts cultured with or without BMP-2. *Mct1* knockdown reduced ALP activity in primary cultures of osteoblasts even in the presence of BMP-2 (Fig. 2a,b), while it also suppressed BMP-2-induced expression of mRNAs for *Tnap* and *Bglap*. In contrast, *Mct1* siRNA induced a 10% increase in *Runx2* mRNA expression. *Sp7* mRNA expression was not affected by *Mct1* siRNA (Fig. 2c).

### No effects of *Mct1* knockdown on Smad and MAPK pathways in C2C12 cells stimulated by BMP-2

**Figure 3:**
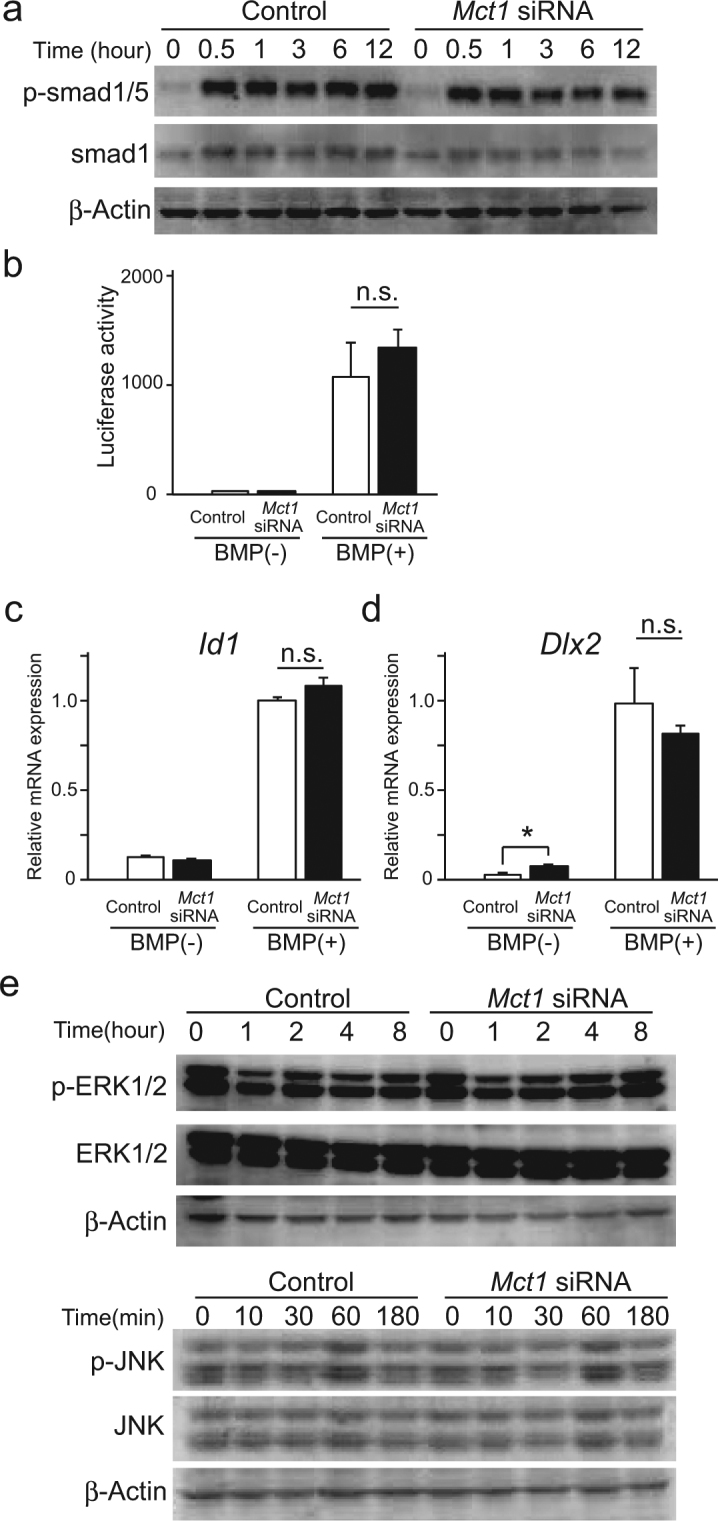
Effects of *Mct1* knockdown on Smad and MAP kinase pathways in C2C12 cells after stimulation by BMP-2. (**a**) Control and *Mct1* siRNA-introduced C2C12 cells were exposed to BMP-2 (300 ng/mL) for the indicated periods. Phosphorylation of Smad1/5 and expression of Smad1 at various times after addition of BMP-2 were evaluated by western blotting. (**b**) C2C12 cells harboring control or *Mct1* siRNA were transfected with an Id1.0-luciferase reporter plasmid and TK vector. After incubation for 24 hours with (+) or without (−) BMP-2 (300 ng/mL), light emission intensity was measured. Relative firefly luciferase reaction values were normalized against that of the luminescent reaction of Renilla luciferase from the TK vector. (**c**,**d**) C2C12 cells harboring control or *Mct1* siRNA were incubated for 24 hours with (+) or without (−) BMP-2 (300 ng/mL). Expressions of *Id1* mRNA (**c**) and *Dlx2* mRNA (**d**) were quantitatively assessed by real-time RT-PCR, then normalized against that of *Gapdh*, with the results shown as relative values. (**b**,**c**) Data are expressed as the mean ± SD (n = 3–5). n.s., not significant. (**e**) Control and *Mct1* siRNA-introduced C2C12 cells were exposed to BMP-2 (300 ng/mL) for the indicated periods, then expressions of ERK1/2 and JNK, as well as their phosphorylation were evaluated by western blotting. (**a**,**e**) Representative data from 3 independent experiments are shown. Quantitative evaluations of phosphorylation of Smad1/5, ERK1/2, and JNK are shown in Supplementary Figure S3. Full-length blots are presented in Supplementary Figure S9.

**Figure 4:**
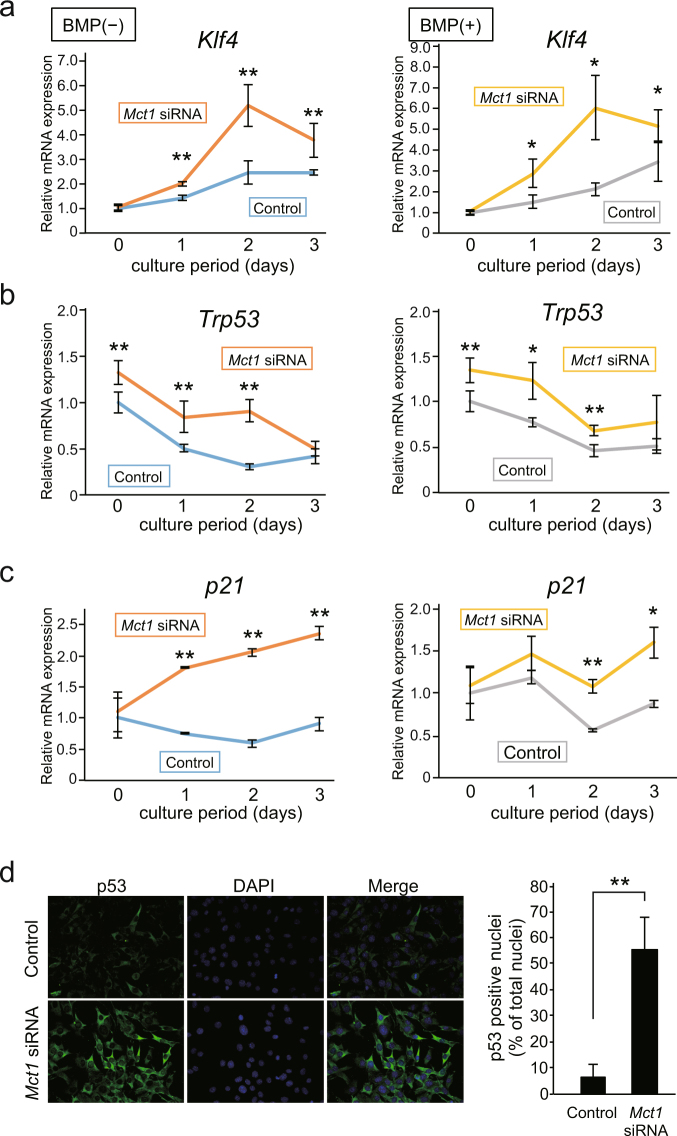
Induction of expression and activation of p53 in C2C12 cells by *Mct1* knockdown. (**a**–**c**) Control and *Mct1* siRNA-introduced C2C12 cells were cultured for the indicated periods in the absence (−) or presence (+) of BMP-2 (300 ng/mL). Expressions of mRNAs for *Klf4* (**a**), *Trp53* (**b**), and *p21* (**c**) were analyzed by real-time RT-PCR, then normalized against that of *Gapdh*, with the results indicated as relative values. (**a**–**c**) Values are expressed as the mean ± SD (n = 3–5). (**d**) C2C12 cells were introduced with control or *Mct1* siRNA, then p53 protein was detected using an immunocytochemical method. Nuclei were stained with DAPI. (**e**) Ratios of p53-positive nuclei in cultures of C2C12 cells introduced with control or *Mct1* siRNA. (**a**–**c**,**e**) Data are expressed as the mean ± SD (n = 3–5). **Significantly different from control group (*p < 0.05, **p < 0.01).

To elucidate the mechanism by which *Mct1* knockdown suppresses osteoblast differentiation of C2C12 cells induced by BMP-2, we investigated its effects on the downstream signaling pathways of BMP-2^11^. Initially, we examined the effect of *Mct1* knockdown on the Smad pathway. Phosphorylation of Smad1/5 after addition of BMP-2 was not affected by introduction of *Mct1* siRNA (Fig. 3a, Suppl. Fig. S3a). Although *Id1* is well known as a target gene of Smad1/5/8, introduction of *Mct1* siRNA did not have an effect on BMP-2-induced expression of luciferase activity in C2C12 cells transfected with the *Id1* promotor-coupled luciferase gene (Fig. 3b) or mRNA expression of intrinsic *Id1* induced by BMP-2^12^ (Fig. 4c). Furthermore, while *Mct1* knockdown up-regulated the mRNA expression of *Dlx2*, another target gene of BMP/Smad pathway^13^, in the absence of the cytokine, it did not have effects on BMP-2-induced expression of *Dlx2* (Fig. 3d). These results indicated that reduced *Mct1* expression downregulates osteoblastic differentiation via mechanisms other than modulation of the Smad pathway. Next, we examined the effect of *Mct1* knockdown on activation of the MAP kinase pathway, which is known to positively regulate osteoblast differentiation^14^. Introduction of *Mct1* siRNA did not have effects on phosphorylation of ERK1/2 or JNK (Fig. 3e, Suppl. Fig. S3b,c). The PI3K/AKT pathway is also known to induce osteoblast differentiation under stimulation by BMP-2^15^. However, *Mct1* knockdown only slightly augmented the phosphorylation of AKT, while BMP-2 did not have an effect on activation of AKT in *Mct1*-silenced C2C12 cells (Suppl. Fig. S4a,b). These effects of *Mct1* knockdown on AKT phosphorylation cannot explain the suppressed osteoblastic differentiation of C2C12 cells introduced with *Mct1* siRNA. We concluded that the MAP kinase pathway is not a major point of action of MCT-1 for promotion of BMP-2-induced osteoblast differentiation.

### *Mct1* knockdown enhanced expression of *Trp53* and *Klf4*

It has been reported that a reduced concentration of serum in culture medium induces myogenic differentiation of C2C12 cells in the absence of BMP-2^7^. However, the role of MCT-1 in myogenic differentiation has not been described, thus we analyzed the effects of *Mct1* knockdown on differentiation of C2C12 cells into myocyte-like cells. The expression of mRNAs for *MyoD*, a myocyte marker gene^16,17^, was not significantly affected by introduction of *Mct1* siRNA in C2C12 cells (Suppl. Fig. S5). On the other hand, that of *Myogenin*, another myocyte marker gene, was up-regulated by co-transfection of *Mct1* siRNA and *Trp53* siRNA. It was previously reported that activation of KLF4 by ERK5 promoted myotube formation in cultures of C2C12 cells^18^, while the present results showed that the expression of *Klf4* mRNA in C2C12 cells following introduction of *Mct1* siRNA was increased in both the absence and presence of BMP-2 (Fig. 4a). It has also been shown that KLF4 directly binds to RUNX2 and inhibits its transcriptional activity^19^. However, *Mct1* knockdown suppressed the expression of *Runx2* in C2C12 cells (Fig. 1c), indicating that increased *Klf4* gene expression is not the sole mechanism for suppression of differentiation of C2C12 cells into osteoblast-like cells. Nevertheless, we noted increased expression of mRNA for the p53 gene *Trp53* (Fig. 4b).

In order to clarify the relationship between *Trp53* and *Klf4* in *Mct1* siRNA-introduced C2C12 cells, a time course study of the expression of each gene was performed. An increase in expression of *Trp53* in *Mct1* siRNA-introduced C2C12 cells was observed prior to induction of differentiation caused by lowering the concentration of serum in both the presence and absence of BMP-2 (Fig. 4b). On the other hand, as shown in Fig. 4a, increased expression of *Klf4* mRNA was observed at least 24 hours after induction of both myogenic and osteoblastic differentiation by cultivation of C2C12 cells in the absence and presence of BMP-2. It was previously reported that p53 induces the expression of *Klf4*^20^, thus it is plausible that *Trp53*, whose expression was shown to be augmented by *Mct1* knockdown, induces *Klf4* expression. We confirmed functional increments of p53 and KLF4 in *Mct1*-silenced C2C12 cells by enhanced expression of *p21* (Fig. 4c), one of the target genes of both p53 and KLF4^20^. Furthermore, fluorescent immunostaining of p53 revealed that both p53 protein expression and its accumulation in nuclei were enhanced in *Mct1*-silenced C2C12 cells (Fig. 4d).

### MCT-1 positively regulated osteoblastic differentiation of C2C12 cells via down-regulation of p53

**Figure 5:**
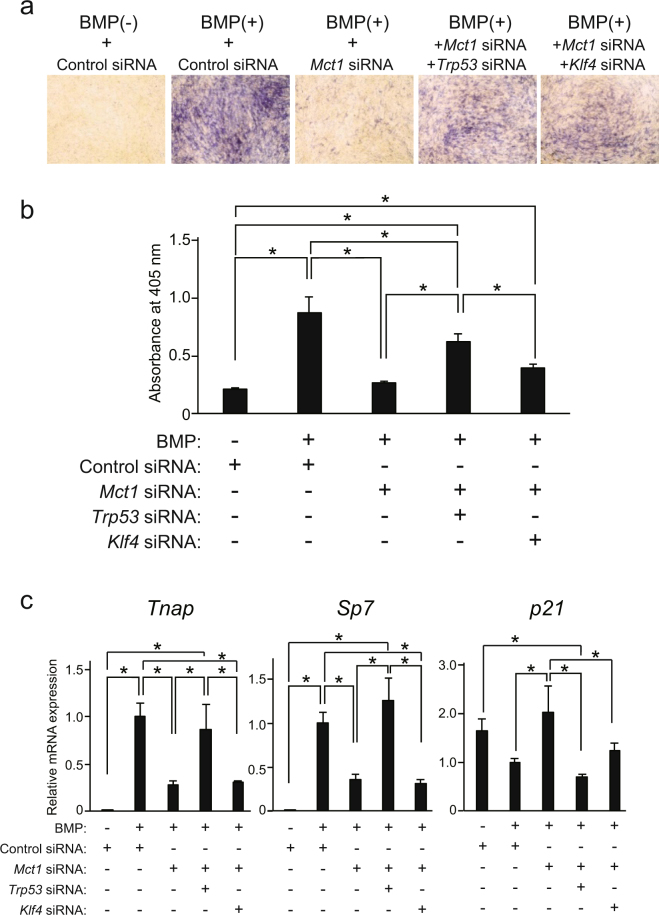
Osteoblastic differentiation suppressed by *Mct1* siRNA recovered by introduction of *Trp53* siRNA. C2C12 cells were introduced with control or *Mct1* siRNA in combination with *Trp53* siRNA or *Klf4* siRNA, then cultured for 72 (**a**) or 48 (**b**) hours in the absence (−) or presence (+) of BMP-2 (300 ng/mL). (**a**) ALP activity in the cultures was detected by staining. (**b**) ALP activity in lysates of C2C12 cells. (**c**) Expression of mRNAs for *Tnap* and *Sp7* were analyzed by real-time RT-PCR, then normalized against that of *Gapdh*, with the results indicated as relative values. (**b**,**c**) Data are expressed as the mean ± SD (n = 3–5). *Significant difference between groups (*p < 0.05).

The suppression of BMP-2-induced ALP activity in C2C12 cells by *Mct1* siRNA was partially recovered by co-introduction of *Trp53* siRNA (Fig. 5a,b), which also led to recovery of suppressed expression of *Sp7* mRNA as well as that of *Tnap* mRNA (Fig. 5c). Together, these results suggested that *Mct1* knockdown induces increased expression of p53, leading to suppressed osteoblastic differentiation of C2C12 cells in the presence of BMP-2. On the other hand, *Klf4* siRNA did not have significant effects on ALP activity lowered by *Mct1* siRNA (Fig. 5a,b) nor the expression of *Tnap*, *Sp7*, or *p21* in *Mct1*-silenced C2C12 cells.

## Discussion

Although it has been widely reported that MCT-1 plays important roles in cellular energy metabolism^1–3^, other biological roles of this transporter have rarely been examined. In the present study, we found two novel functions, requirement of MCT-1 for osteoblast differentiation, and its activity to suppress expression and activation of p53. Differentiation of myoblastic C2C12 cells into osteoblast-like cells following exposure to BMP-2 is regarded as a model of osteoblast differentiation from cells in a lower stage of differentiation, such as mesenchymal stem cells^21^. Using this model, we found that *Mct1* knockdown suppressed osteoblastic differentiation of C2C12 cells.

We also noted that *Mct1* siRNA suppressed the expressions of *Tnap* and *Bglap*, as well as ALP activity in not only C2C12 cells but also mouse primary osteoblasts (Fig. 2a–c). Although *Mct1* siRNA induced a 10% increase in expression of *Runx2* mRNA, that limited escalation of expression did not alter the expression of *Sp7* (Fig. 2c). Since *Mct1* knockdown suppressed the expression of *Tnap* and *Bglap*, late-phase marker genes of osteoblasts, without suppression of *Runx2* or *Sp7* expression in differentiated osteoblasts, it is possible that MCT-1 promotes expression of these late-phase differentiation markers of osteoblasts by regulating other transcription factors or the signaling system independent of induction of RUNX2 and Sp7^22^.

We also did not find a noticeable effect of *Mct1* siRNA on well-known events downstream of stimulation by BMP-2, namely, expression of the *Id1* gene or phosphorylation of Smad-1/5, ERK, JNK, and AKT (Fig. 3, Suppl. Figs S3 and S4). These results suggested that *Mct1* knockdown suppresses osteoblast differentiation of C2C12 cells in a BMP-2-independent manner. It is interesting that *Mct1* siRNA augmented the expressions of *Trp53*, *Klf4*, and *p21* in C2C12 cells in both the presence and absence of BMP-2 (Fig. 4a–c), indicating that MCT-1 suppresses expression of those genes independent of BMP-2. Our finding of increased expression of *p21* mRNA in *Mct1*-silenced C2C12 cells is consistent with induction of *Trp53* expression, since *p21* is one of the genes targeted by p53^23^. It is well known that p53 suppresses cell proliferation by induction of expression of p21, a cyclin-dependent kinase inhibitor, while another study reported that galectin-3 stabilizes p21 protein in prostate cancer cells^24^. It is possible that p21instability explains why the augmented expression of mRNAs for *Trp53* and *p21* did not have an effect on proliferation of C2C12 cells harboring *Mct1* siRNA.

The well-known tumor suppressor and transcription factor p53 has a diverse array of biological functions, among which DNA repair, regulation of cell cycle and senescence, and induction of apoptosis are regarded as the most important^25^. In addition, reports have shown a role of p53 in differentiation of various types of cells^26^, such as osteoblasts, in which p53 suppresses transcription of the *RUNX2* gene^27^. It is also known that glycolysis is very active in tumor cells even in aerobic conditions, resulting in acidification of tumor tissues by enhanced production and excretion of lactate^28^. Upregulated expression of MCT-1 in p53-null and -mutated tumor cells has also been reported^29,30^. On the other hand, regulation of p53 expression by MCT-1 has not been previously examined. The present findings showed that *Mct1* knockdown enhanced the expression of *Trp53* (Fig. 4), indicating that MCT-1 is a negative regulator of p53 expression.

KLF4 is also known as a multifunctional protein and has been shown to be an essential factor for induction of pluripotent stem cells^31,32^, while another report noted that KLF2 and KLF4 function as positive regulators of myotube formation by C2C12 cells^18^. In our experimental settings, the effect of *Mct1* knockdown on expression of *Myogenin*, a marker gene of myogenic differentiation, was small and insignificant, and co-transfection of *Klf4* siRNA also did not have a significant effect on expression of *Myogenin* mRNA in *Mct1*-silenced C2C12 cells, either (Suppl. Fig. S5). Therefore, enhanced expression of *Klf4* in *Mct1*-silenced C2C12 cells (Fig. 4a) does not have an appreciable role in differentiation of C2C12 cells into myocyte-like cells (Suppl. Fig. S5). On the other hand, introduction of *Trp53* siRNA significantly enhanced expression of the *Myogenin* gene in *Mct1*-silenced C2C12 cells (Suppl. Fig. S5), indicating that p53 has an ability to suppress myogenic differentiation in C2C12 cells.

A recent report described incorporation of lactate through MCT-1-stabilized HIF-1α, which caused promotion of osteoblast differentiation^33^. It has also been reported that HIF-1α enhanced the expression of *Trp53*^34^. To examine if HIF-1α has an important role in induction of *Trp53* in our experimental settings, we performed immunostaining of C2C12 cells for HIF-1α with or without introduction of *Mct1* siRNA. However, *Mct1* siRNA did not have a noticeable effect on either the expression or nuclear accumulation of HIF-1α protein in C2C12 cells following stimulation with BMP-2 (Suppl. Fig. S6b). Thus, it seems unlikely that HIF-1α plays an important role in the induced expression of *Trp53* after *Mct1* knockdown. On the other hand, in the absence of BMP-2, cytoplasmic HIF-1α expression was increased by *Mct1* siRNA (Suppl. Fig. S6a). It is well known that the protein level of HIF-1α is up-regulated by hypoxia, and it has also been shown that transcription of the *HIF1A* gene is promoted by activation of the PI3K-Akt and MAPK pathways^35,36^. In our experiments, *Mct1* siRNA did not alter nuclear accumulation of HIF-1α, whereas phosphorylation of Akt was augmented in *Mct1*-silenced C2C12 cells even in the absence of BMP-2 (Suppl. Fig. S4). Therefore, it is possibile that decreased expression of MCT-1 stimulates the Akt pathway and enhances HIF-1α expression.

Another study showed that aerobic glycolysis suppresses p53 activation in cancer cells^37^. In the present experiments, the amount of intracellular lactate was found to be increased in *Mct1*-silenced C2C12 cells (Suppl. Fig. S1c), indicating a decrease in excretion of lactate via MCT-1 in those cells. Since lactate dehydrogenase catalyzes both the reduction of pyruvate into lactate and oxidation of lactate into pyruvate, it can be speculated that lactate accumulation in cytosol suppresses the former reaction and facilitates the latter, which might reduce the efficacy of glycolysis by lowering the NAD^+^/NADH ratio. We observed reduced amounts of incorporated L-glucose in *Mct1* siRNA-introduced C2C12 cells when cultured in differentiation-inducing medium with a lower serum concentration (2.5%) (Suppl. Fig. S7). In addition, osteoblastic differentiation of C2C12 cells when cultured in DMEM containing a low level of L-glucose was suppressed (Suppl. Fig. S8). Therefore, suppression of glycolysis may promote p53 activation in and osteoblastic differentiation of *Mct1*-silenced cells.

In conclusion, we found that MCT-1 facilitates osteoblast differentiation via suppression of p53 expression and activation, thus knockdown of the *Mct1* gene resulted in increased p53 activity, which induced the expression of *Klf4* and *p21*. We speculate that the p53 pathway is involved in inhibition of *Runx2* and *Sp7* expression, as well as expression of osteoblast marker genes, such as *Tnap* and *Bglap*.

## Methods

### Ethical approval statement

All experimental protocols were approved by Showa University Animal Experiment Facility and Showa University Genetic Recombination Laboratory (Approval number: 17053). We confirm that all experiments were performed in accordance with the relevant guidelines and regulations.

### Reagents

Recombinant human BMP-2 was obtained from R&D Systems (Minneapolis, MN, USA). AZD3965, a specific MCT-1 inhibitor, was purchased from Cayman Chemical (Ann Arbor, MI, USA). Antibodies against smad1, phospho-smad1/5, ERK1/2, phospho-ERK1/2, ERK5, phospho-ERK5, JNK, phospho-JNK, p38MAPK, phospho-p38MAPK, AKT, and phosphor-AKT were obtained from Cell Signaling Technology (Beverly, MA, USA). Antibodies against myosin heavy chain (MF-20), p53, and HIF-1α were obtained from Developmental Studies Hybridoma Bank (Iowa City, IA, USA), Abcam (Cambridge, UK), and GeneTex, Inc. (Irvine, CA, USA), respectively.

### Cell cultures

C2C12 cells were purchased from RIKEN BioResource Center (C2C12 #RCB0987, Tsukuba, Japan) and grown in Dulbecco’s modified Eagle’s medium (DMEM, Wako Pure Chemical Industries, Osaka, Japan) supplemented with 15% fetal bovine serum (FBS). To induce differentiation into osteoblast-like cells, the medium was replaced with DMEM supplemented with 2.5% FBS and 300 ng/mL BMP-2, while myotube formation was induced by replacing the medium with DMEM supplemented with 2.5% FBS. Mouse primary osteoblasts were isolated from calvaria of neonatal ddY mice and grown in αMEM (Wako Pure Chemical Industries) supplemented with 10% FBS. Osteoblast phenotypes were induced by stimulation with 10% FBS and 300 ng/ml BMP-2 in the same medium. Stealth^TM^ siRNAs (Invitrogen, Carlsbad, CA, USA) for mouse *Mct1*, *Trp53*, and *Klf4*, and negative control siRNA were introduced into 40–50% confluent cells using Lipofecttamine^TM^ RNAiMAX (Invitrogen) by reverse transfection. BMP-2 was added to the cultures at 24 hours after siRNA introduction.

### Real-time RT-PCR

Total RNA was extracted from cells using TRIzol reagent (Invitrogen) according to the manufacturer’s instructions. Reverse transcription reactions were performed using ReverTra ACE RT qPCR master Mix (TOYOBO Co. Ltd., Osaka, Japan). Quantitative real-time RT-PCR was performed using a TaqMan^TM^ Gene Expression Assay (Applied Biosystems, Carlsbad, CA, USA). The assay IDs of the genes were as follows: *Gapdh*, Mm99999915_g1; *Mct1*, Mm01306379_m1; *Mct2*, Mm00441442_m1; *Mct3*, Mm00445115_m1; *Mct4*, Mm00446102_m1; *Tnap*, Mm00475834_m1; *Runx2*, Mm00501584_m1; *Sp7*, Mm04209856_m1; *Bglap*, Mm03413826_mH; *Dlx2*, Mm00438427_m1; *Trp53*, Mm01731290_g1; *p21*, Mm00432448_m1; *Klf4*, Mm00516104_m1; and *Id1*, Mm00775963_g1. Amplification signals from the target genes were normalized against that of *Gapdh*.

### Cell proliferation assay

C2C12 cells were plated at a density of 1 × 10^4^ cells/well in 96-well plates, and cultured in the presence or absence of BMP-2 (300 ng/mL) for various periods up to 72 hours. At the end of each culture period, cells were incubated for 1 hour with solution from a CellTiter 96^®^ Aqueous One Solution cell proliferation assay kit (Promega, Madison, WI, USA), then MTS-formazan was determined by reading absorbance at 490 nm.

### Determination of intracellular lactate

C2C12 cells were plated at a density of 5 × 10^5^ cells/well in 6-well plates and cultured for 24 hours, then washed with PBS and homogenized in 1% Nonidet P-40 (200 μL) under sonication on ice. Lactate concentrations in the cell lysates were determined using F-kit D-lactic acid/L-lactic acid (J.K. International, Tokyo, Japan), according to the manufacturer’s instructions.

### ALP activity staining

Cells were plated in 96-well plates at a density of 1 × 10^4^ cells/well, and cultured for 72 hours in the presence or absence of BMP-2 (300 ng/mL). Cells were fixed for 30 minutes in 4% paraformaldehyde and washed with PBS, then incubated for 30 minutes at 37 °C with 100 mmol/L Tris-HCl buffer (pH 8.5) containing 270 μmol/L naphthol AS-MX phosphate (Sigma-Aldrich) and 1.4 mmol/L Fast blue BB (Sigma-Aldrich). After washing with tap water, they were observed under a microscope.

### Determination of ALP activity

Cells were cultured in the same conditions as described in the ALP activity staining subsection above, then washed with PBS and homogenized with 1% Nonidet P-40 (50 μL) under sonication on ice. Cell lysates (10 μL) were added to 50 μL of 0.2 mol/L Tris–HCl buffer (pH 9.5) containing 1 mmol/L MgCl_2_ and 12.5 mmol/L disodium *p*-nitrophenyl phosphate (Wako Pure Chemical Industries). After incubation for 15 minutes at 37 °C, reactions were terminated by addition of 50 μL of 0.5 mol/L NaOH and absorbance of the reaction mixture at 405 nm was read using a micro-plate reader (SH-1000, Corona Electric, Ibaraki, Japan). The increase in absorbance in after 15 minutes was divided by the amount of cellular protein and the obtained value was used to express the specific activity of ALP.

### Alizarin red staining

Cells were plated in 96-well plates at a density of 1×10^4^ cells/well, and cultured for 4 days in the medium described above in the presence or absence of BMP-2 (300 ng/mL), followed by additional 3-day culture in DMEM supplemented with 2.5% FBS, ascorbic acid (50 μg/mL), β-glycerophosphate (10 mmol/L), and dexamethasone (10 nmol/L). Cells were washed with PBS, fixed in 95% methanol, stained with 1% alizarin red S (pH 6.3–6.5) (Wako Pure Chemicals) for 5 minutes, and washed with water to remove unbound dye. Calcified nodules visualized by alizarin red S were observed under a microscope. For evaluation of calcium deposition, alizarin red S bound to cell matrices was dissolved in 10% (w/v) cetylpyridinium chloride in water and quantified by measuring absorbance at 570 nm.

### Immunostaining of myosin heavy chain protein

C2C12 cells were plated in 96-well plates at a density of 1 × 10^4^ cells/well and cultured for 6 days. Next, they were fixed for 30 minutes in 4% paraformaldehyde, then washed with PBS, permeated with 0.1% Triton X-100 in PBS for 5 minutes, and incubated overnight at 4 °C with 0.5 μg/mL of a mouse anti-αMHC monoclonal antibody in PBS containing 10% rabbit serum. After washing again with PBS, cells were incubated for 30 minutes with Histofine^®^ Simple Stain^TM^ MAX PO (MULTI) (Nichirei. Co., Tokyo, Japan). Finally, the reaction products were visualized using a Histofine AEC substrate kit (Nichirei Co., Tokyo, Japan).

### Immunostaining of p53 and HIF-1α

C2C12 cells were fixed for 20 minutes in 4% paraformaldehyde at room temperature, then permeated with 0.1% Triton X-100 in PBS for 5 minutes. Next, they were incubated overnight at 4 °C with 0.5 μg/mL of the anti-p53 antibody or anti-HIF-1α in PBS containing 10% rabbit serum, followed by incubation for 45 minutes at room temperature with an Alexafluor-488-conjugated secondary antibody (Invitrogen) diluted in PBS (1:100). Nuclei were stained with 4′,6′-diamidine-2′-phenylindole dihydrochloride (DAPI) and fluorescence was observed using a BZ-9000 microscope (KEYENCE, Osaka, Japan).

### Western blot analysis

C2C12 cells were lysed in 10 mmol/L Tris-HCl (pH 7.8) with 1% Nonidet P-40, 0.15 mol/L NaCl, and a protease inhibitor mixture containing EDTA (Roche Applied Science, Penzberg, Germany). Cell lysates (5 μg of protein) were subjected to SDS-PAGE (10% polyacrylamide gel) under a reducing condition. Following electrophoresis, proteins were transferred onto PVDF membranes and incubated overnight at 4 °C with the primary antibodies against smad1, phospho-smad1/5, ERK1/2, phospho-ERK1/2, ERK5, phospho-ERK5, JNK, phospho-JNK, p38 MAPK, phospho-p38 MAPK, AKT, and phosphor-AKT, followed by incubation with horseradish peroxidase-conjugated anti-rabbit IgG (GE Healthcare, Little Chalfont, UK). Immunoreactive bands were visualized by an enhanced chemiluminescence reaction with an ECL Prime Western Blot Detection System (GE Healthcare). Intensity of the chemiluminescent bands was quantitatively analyzed using Versa Doc 5000 MP (Bio-Rad Laboratories, Hercules, CA, USA). The ratio for intensity of a band for phosphorylated protein to that for total protein was calculated.

### Luciferase assay

C2C12 cells were plated in 24-well plates at a density of 1 × 10^4^ cells/well and transfected with an Id1.0-luc reporter plasmid and TK vector (Promega, Madison, WI, USA). After 24 hours of incubation in the presence or absence of BMP-2 (300 ng/ml), luminescence was detected using a Dual-Luciferase Reporter Assay System (Promega). The relative activity of firefly luciferase was obtained after normalization against the luminescent reaction of Renilla luciferase encoded by the TK vector. A pGL4 [luc2/Neo] vector was used as the negative control.

### Statistical analysis

Values are expressed as the mean ± SD. Student’s *t*-test was used to compare results between 2 groups. One-way ANOVA with post-hoc Tukey test was performed for comparing results from 3 or more groups. A *p*-value less than 0.05 was considered to indicate statistical significance.

### Data availability statement

The datasets generated and analyzed for the current study are available from the corresponding author upon reasonable request.

## Electronic supplementary material

Supplementary Information
