# Supplementary material for: Monocarboxylate transporter-1 promotes osteoblast differentiation via suppression of p53, a negative regulator of osteoblast differentiation
Source: Sci Rep. 2018 Jul 12;8:10579. doi: 10.1038/s41598-018-28605-5 (PMC6043614; doi:10.1038/s41598-018-28605-5)
Supplement: Supplementary file 1 — Supplementary Information [file 41598_2018_28605_MOESM1_ESM.pdf]

Supplementary Information

Monocarboxylate transporter-1 promotes osteoblast differentiation via suppression of p53, a negative regulator of osteoblast differentiation

Kiyohito Sasa[1], Kentaro Yoshimura[1*], Atsushi Yamada[1], Dai Suzuki[1], Yoichi Miyamoto[1], Hiroko Imai[1, 2], Kazuhiro Nagayama[1,3], Koutaro Maki[3], Matsuo Yamamoto[2], Ryutaro Kamijo[1]

Departments of [1]Biochemistry, [2]Periodontology, and [3]Orthodontics, Showa University School of Dentistry, Tokyo, Japan

*Corresponding author: Kentaro Yoshimura
Department of Biochemistry, Showa University School of Dentistry, 1-5-8 Hatanodai, Shinagawa, Tokyo 142-8555, Japan
Tel: +81 3 3784 8163, Fax: +81 3 3784 8163, Email: kyoshimura@dent.showa-u.ac.jp

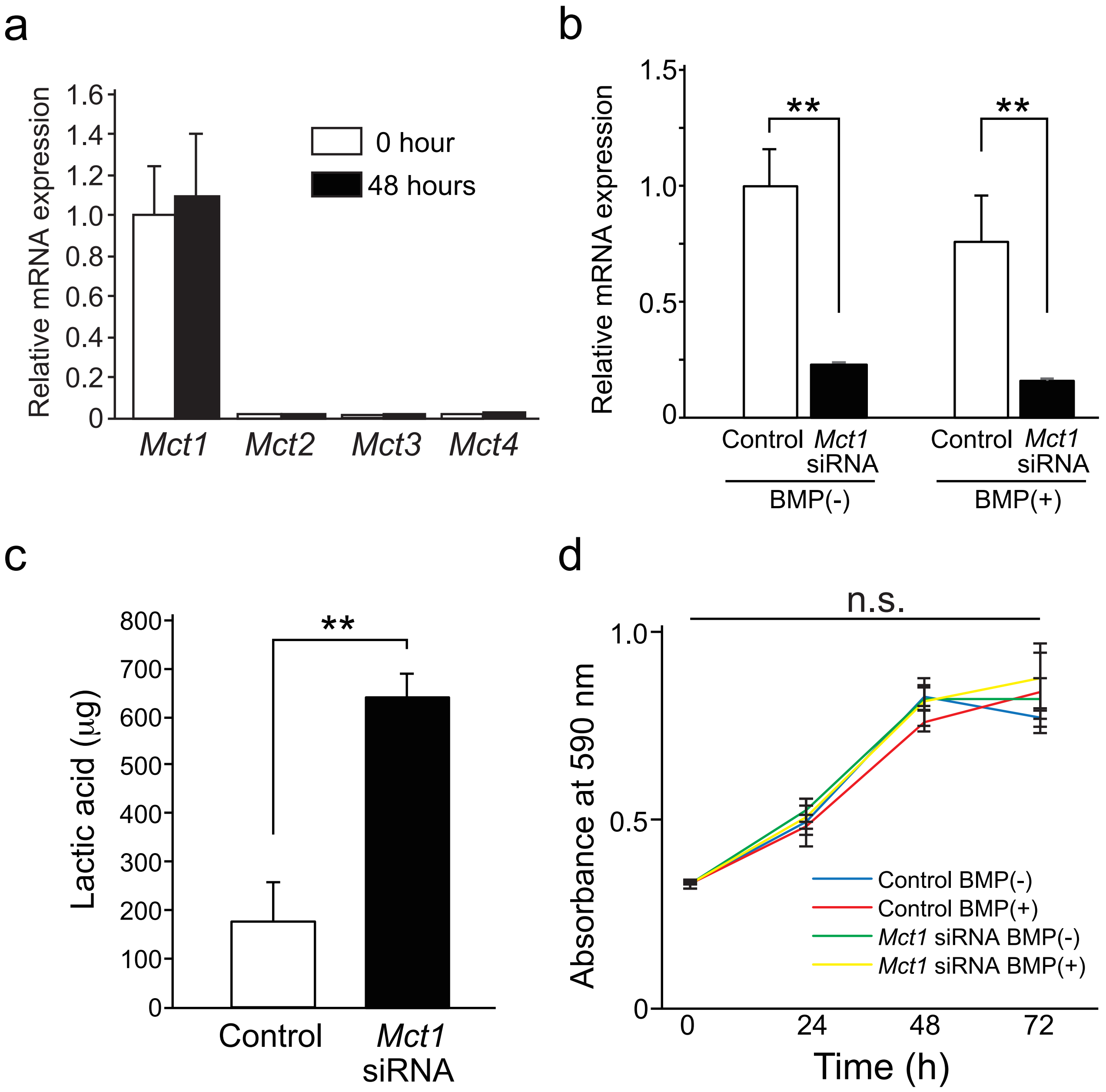

Supplementary Figure S1. Silencing of *Mct1* gene by siRNA in C2C12 cells. (a) C2C12 cells were cultured for the indicated periods. Expression of mRNAs for *Mct1*, *Mct2*, *Mct3*, and *Mct4* were analyzed by real-time RT-PCR. Amplification signals from these genes were normalized against that of *Gapdh*. Relative values are indicated as the mean ± SD (n=3). (b) C2C12 cells were introduced with *Mct1* siRNA and cultured for 48 hours in the presence or absence of BMP-2 (300 ng/mL). *Mct1* gene expression in C2C12 cells was quantitatively assessed by real-time RT-PCR, then normalized against that of *Gapdh*, with the results indicated as relative values. (c) Intracellular lactic acid in C2C12 cells introduced with control or *Mct1* siRNA was determined after culturing for 24 hours. (d) C2C12 cells introduced with control or *Mct1* siRNA were cultured for the indicated periods in the presence or absence of BMP-2 (300 ng/mL). Cell viability was assessed based on the reduction of MTS. (b-d) Values are expressed as the mean ± SD (n=3). **Significantly different from control group (p <0.01). n.s., not significant

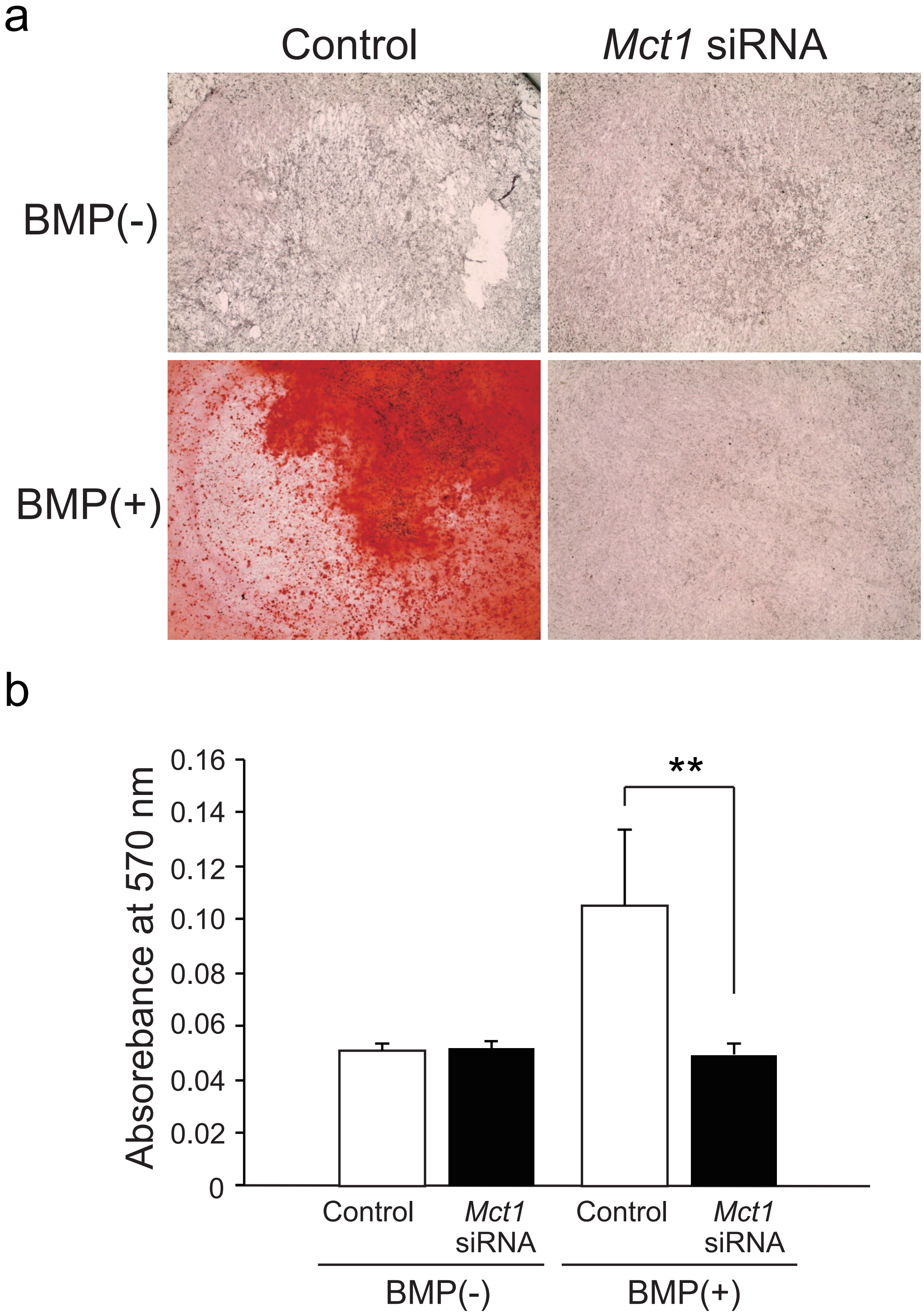

Supplementary Figure S2. Suppression of mineralization by *Mct1* knockdown in C2C12-cell cultures. C2C12 cells introduced with control or *Mct1* siRNA were cultured for 4 days in the presence or absence of 300 ng/mL BMP-2 in DMEM plus 2.5% FBS, followed by additional 3-day culture in DMEM supplemented with 50 μg/mL ascorbic acid, 10 mmol/L β-glycerophosphate, and 10 nmol/L dexamethasone. (a) Cells were stained with 1% alizarin red. (b) Alizarin red bound to cultured cells was dissolved in 10% (w/v) cetylpyridinium chloride and quantified by reading the absorbance at 570 nm. Data are expressed as the mean ± SD for 12 experiments. **Significantly different from control group (p <0.01)

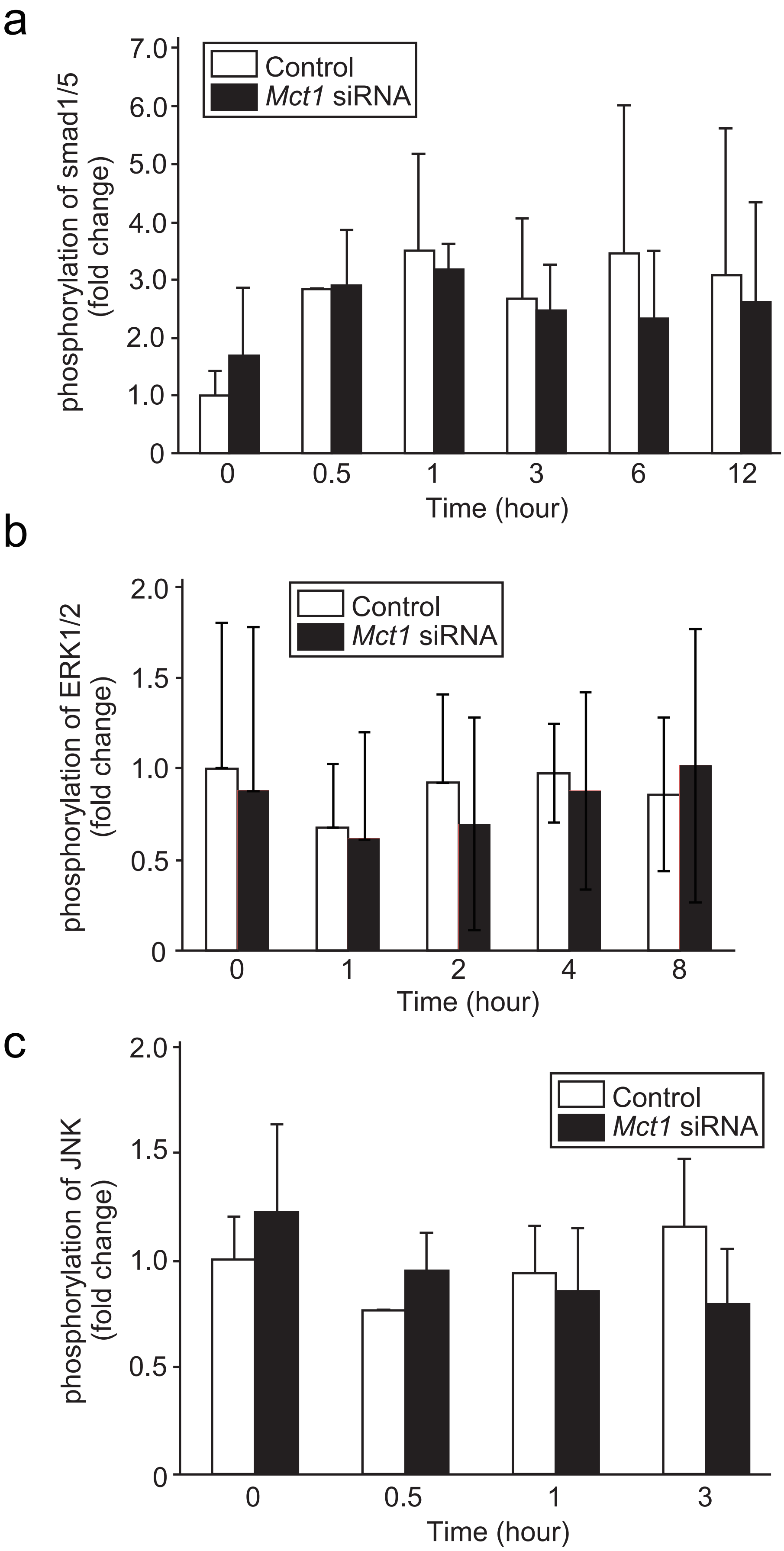

Supplementary Figure S3. Phosphorylation of smad1/5, ERK1/2, and JNK in C2C12 cells after addition of BMP-2. Western blotting for (a) phosphorylated and un-phosphorylated smad1/5, (b) ERK1/2, and (c) JNK of C2C12 cells was performed after incubation with BMP-2 (300 ng/mL) for the indicated periods. Intensity of each band was quantified using Versa Doc 500 MP (Bio-Rad Laboratories). Data are expressed as the mean ± SD for 3 independent experiments. Representative photographs of the membranes are shown in Figure 3.

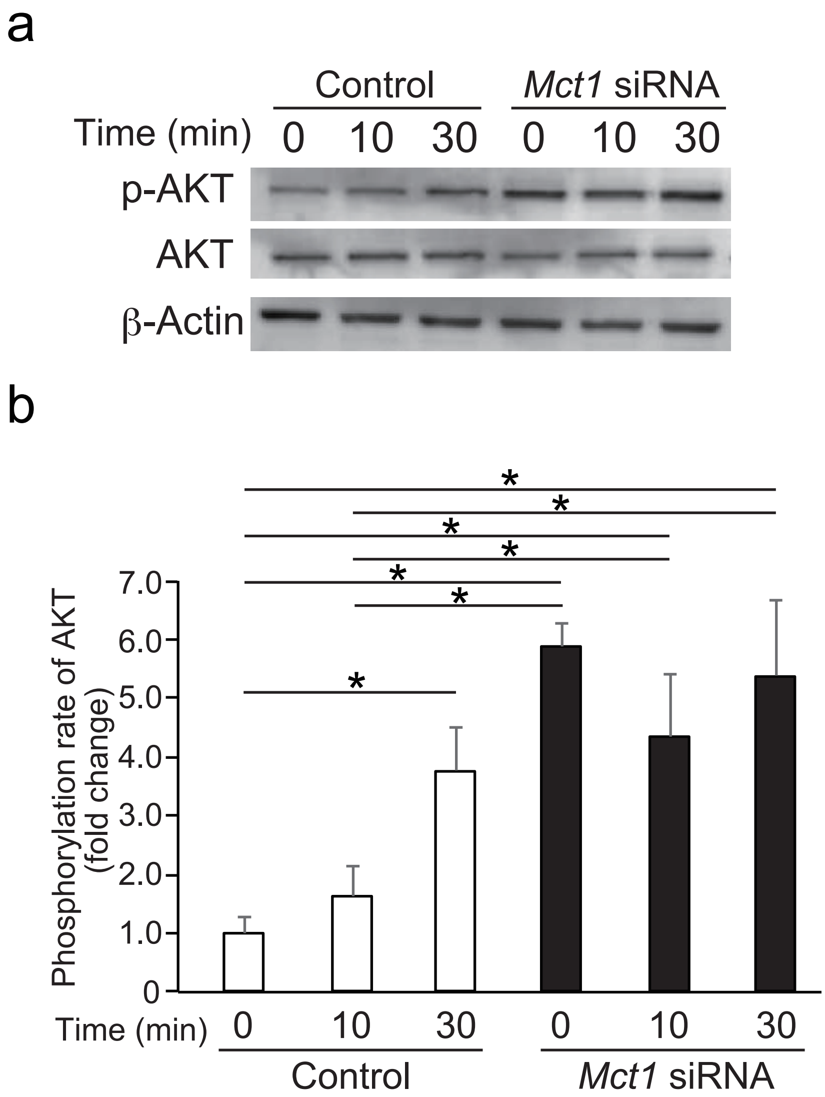

Supplementary Figure S4. Effects of *Mct1* knockdown on phosphorylation of AKT in C2C12 cells after stimulation with BMP-2. (a) C2C12 cells harboring control or *Mct1* siRNA were exposed to BMP-2 (300 ng/mL). AKT and phosphorylated AKT (p-AKT) in cell lysates obtained at the indicated times after addition of BMP-2 were analyzed by western blotting. (b) Quantitative data are expressed as the mean ± SD for 3 independent experiments. *Significant difference between groups (p <0.05)

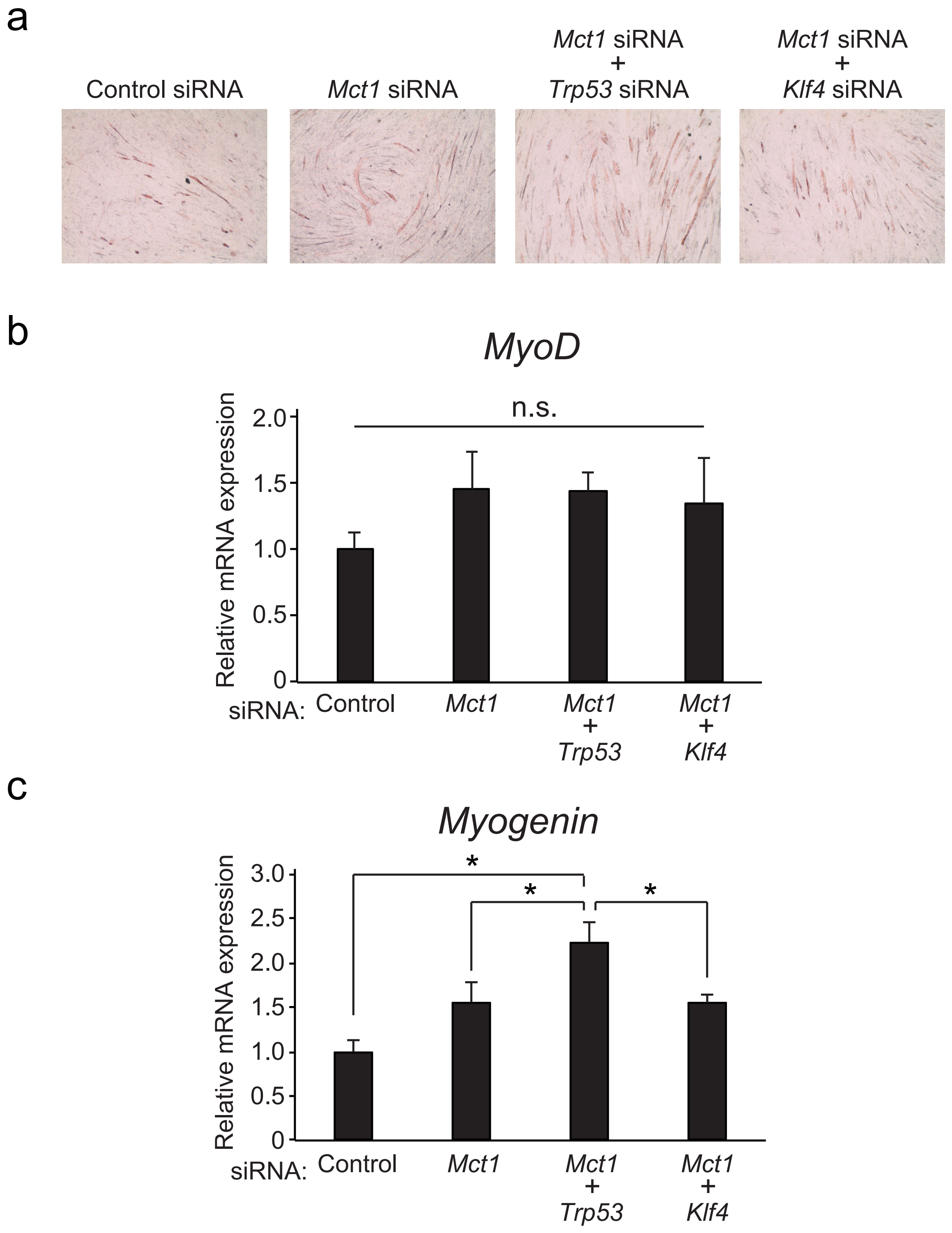

Supplementary Figure S5. Effect of *Mct1* siRNA on myogenic differentiation of C2C12 cells. C2C12 cells introduced with control or *Mct1* siRNA in combination with *Trp53* siRNA or *Klf4* siRNA were cultured for 6 days without addition of BMP-2. (a) Representative results of immunocytochemical analysis of the cells for myosin heavy chain are shown. (b, c) Expression of mRNAs for *MyoD* (b) and *Myogenin* (c) were analyzed by real-time RT-PCR. Amplification signals from these genes were normalized against that of *Gapdh*. Relative values are indicated as the mean ± SD (n=3). *Significant difference between groups (p <0.05)

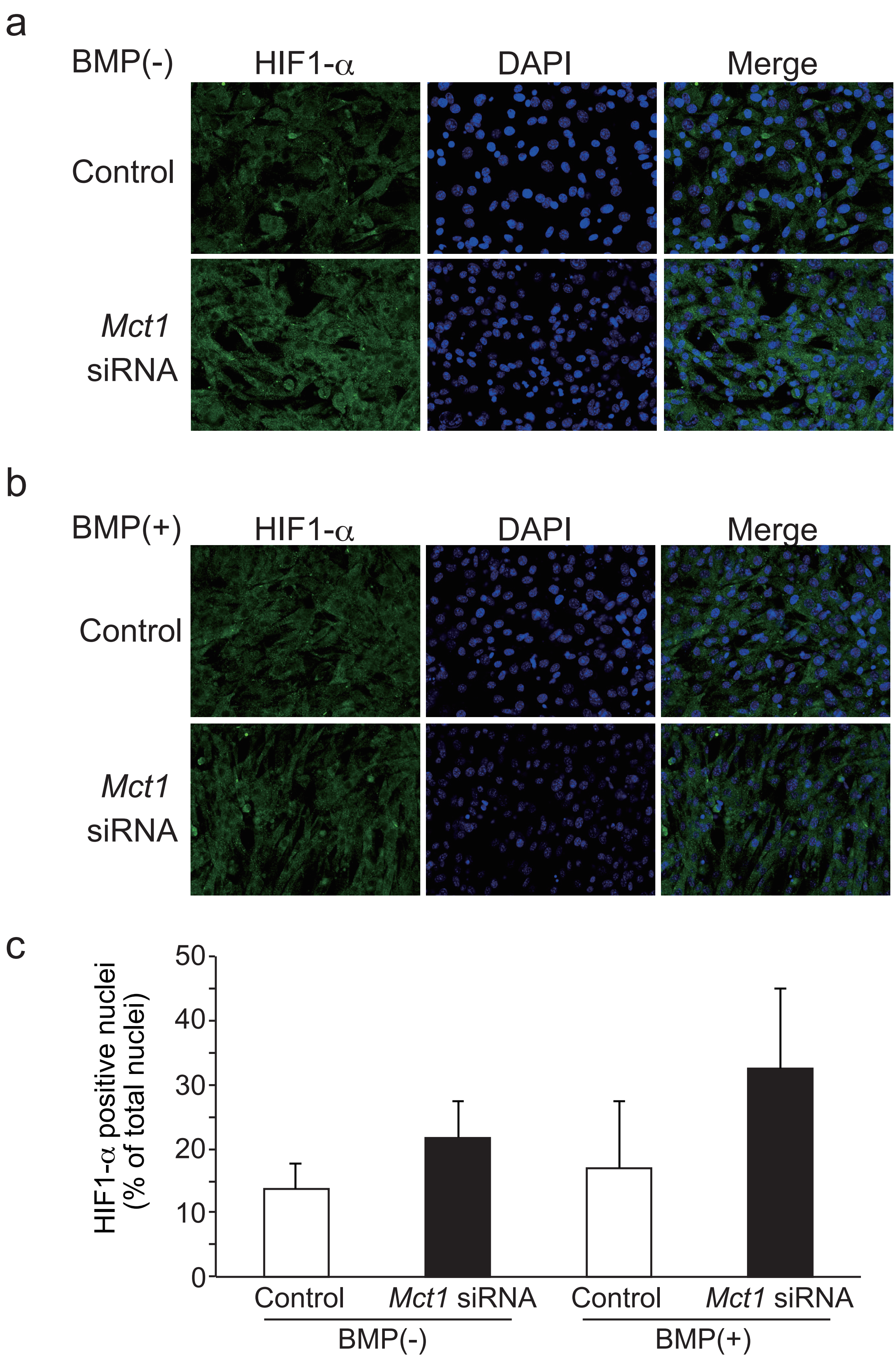

Supplementary Figure S6. Effects of *Mct1* siRNA on expression and nuclear translocation of HIF-1α in C2C12 cells. C2C12 cells introduced with control or *Mct1* siRNA were cultured for 2 days in the absence of BMP-2. (a,b) Representative images of immunofluorescence staining of cells using anti-HIFα (green) and nuclear staining with DAPI (blue) are shown. (c) Quantitative data are expressed as the mean ± SD for 3 independent experiments.

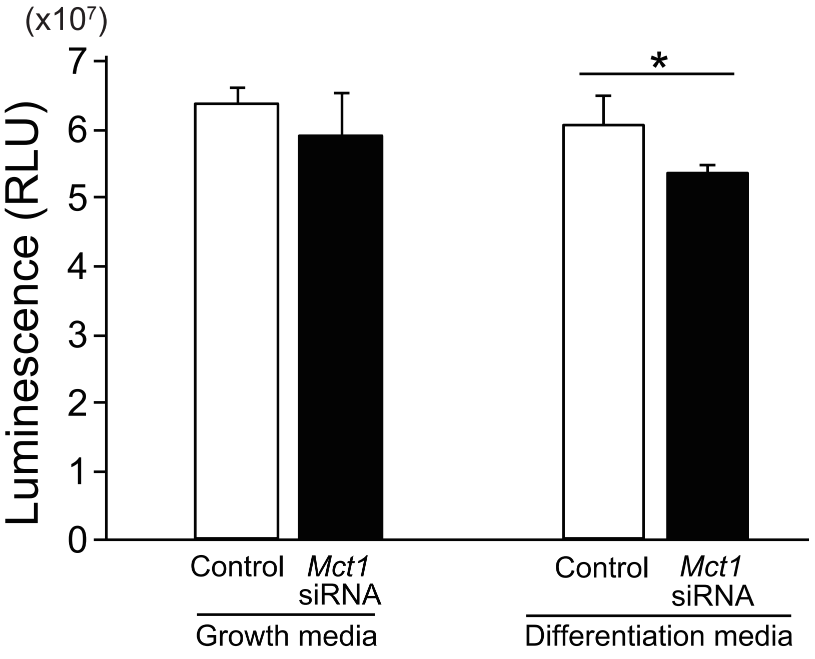

Supplementary Figure S7. Reduction of glucose uptake by *Mct1* siRNA.

Control (Control) or *Mct1* siRNA was introduced into C2C12 cells. After incubation for 2 hours in DMEM supplemented with 2.5% FBS, 2-deoxyglucose uptake was analyzed using a Glucose Uptake-GloTM Assay kit (Promega), with luminescence (vertical axis) proportional to the concentration of 2-deoxyglucose. *Significantly different from control group (p <0.05)

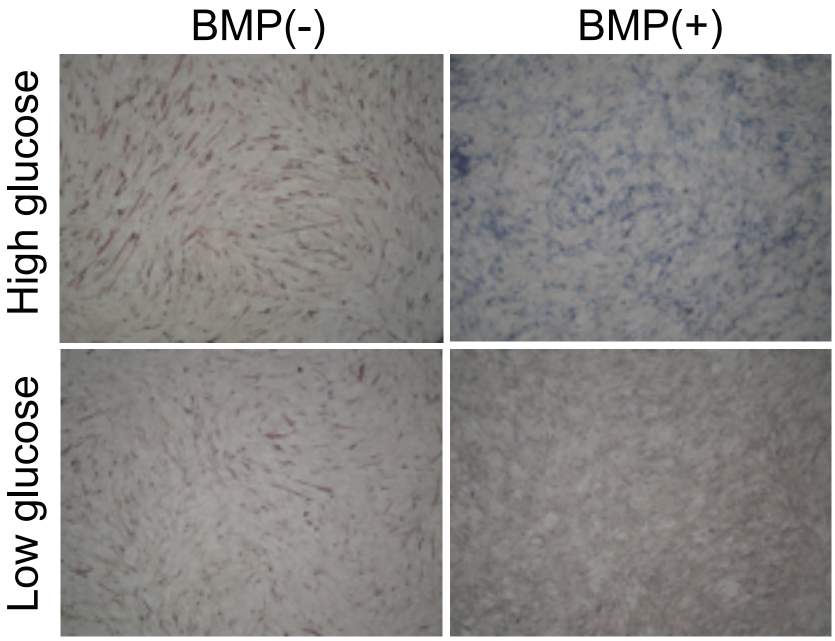

Supplementary Figure S8. Promotion of osteoblast differentiation by glucose in C2C12 cells. ALP activity staining of C2C12 cells cultured in DMEM containing glucose at a concentration of 4500 mg/L (high glucose) or 1000 mg/L (low glucose), and supplemented with 2.5% FBS with (+) or without (-) BMP-2 (300 ng/mL).

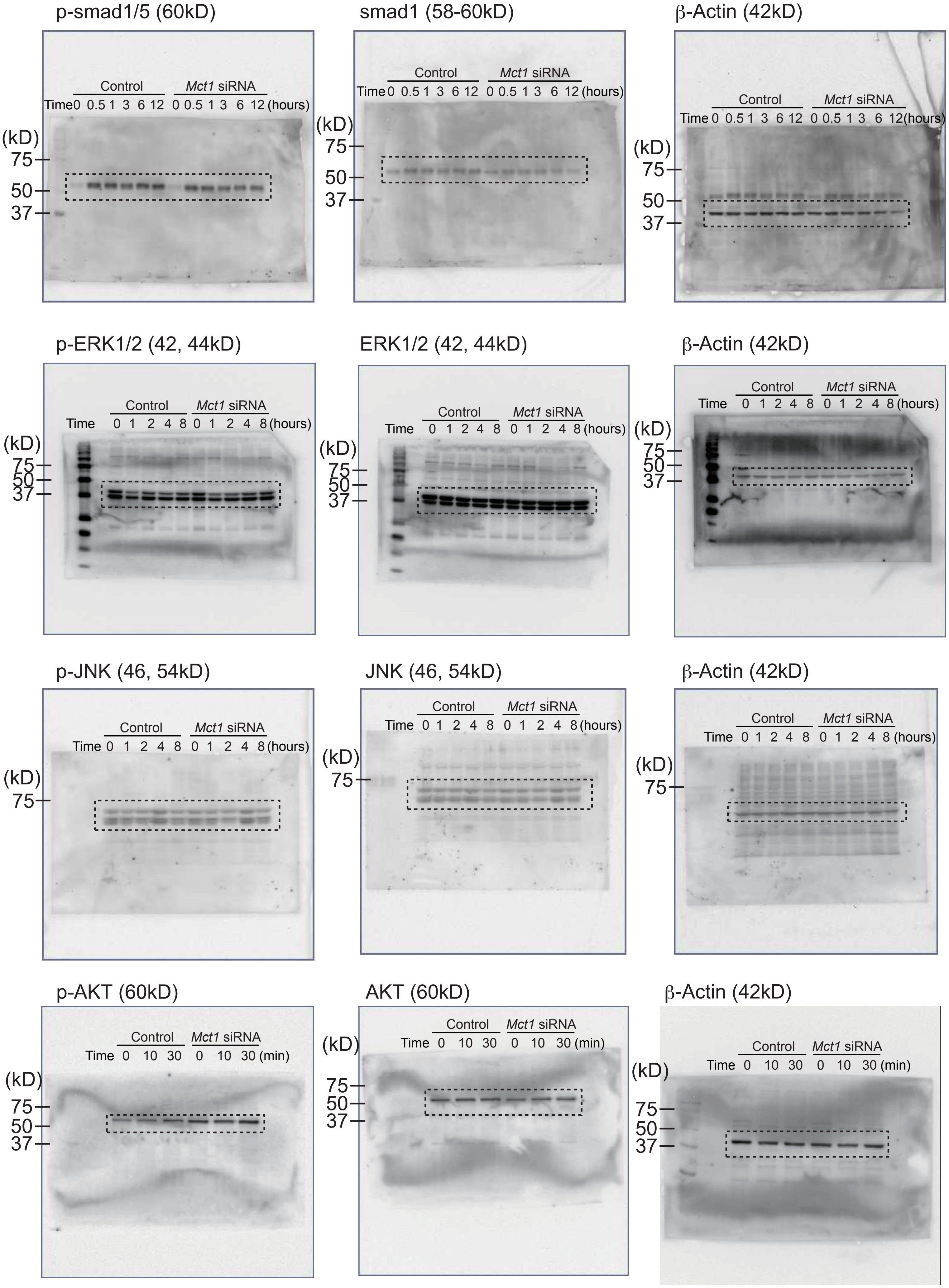

Supplementary Figure S9. Original images of immunoblots with molecular weight standards.
